# Does leukocyte apoptosis play any role in the pathogenesis of experimental pancreatitis?

**DOI:** 10.1186/cc9669

**Published:** 2011-03-11

**Authors:** D Zotos, T Adamis, A Pistiki, K Louis, E Giamarellos-Bourboulis

**Affiliations:** 1 University of Athens, Medical School, Athens, Greece; 2 Attikon University Hospital, Athens, Greece

## Introduction

The role of apoptosis of leukocytes for the final outcome of necrotizing pancreatitis remains to be elucidated.

## Methods

Experimental pancreatitis was induced in rabbits after ligation of the common pancreatic duct. Animals were assigned into sham-operated (group A, *n *= 8) infused 0.3 ml of ethanol 99% above the ligation; into nine infused 0.3 ml of one 10% solution of taurocholic acid above the ligation (group B, *n *= 9); and into 10 infused 0.3 ml of one 20% solution of taurocholic acid above the ligation (group C, *n *= 10). Blood was sampled at serial time intervals; apoptosis of lymphocytes, monocytes and neutrophils was assessed after staining for annexin V and for propidium iodine and flow cytometric analysis. On death or on sacrifice the pancreas was removed. Fat necrosis was assessed by histology; quantitative tissue cultures were done.

## Results

Median survival of group A was 28 days; of group B was 5 days (log-rank vs. group A: 4.155, *P *= 0.042); and of group C was 1.5 days (log-rank vs. group A: 10.356, *P *= 0.001). Mean percentage pancreatic necrosis of groups A, B and C was 2.5, 45.0 and 42.0%, respectively. Respective mean log_10 _of bacteria in the liver was 1.00, 3.13 and 2.48 cfu/g; in the lung 1.26, 2.90 and 2.56 cfu/g; in the spleen 1.00, 3.72 and 2.37 cfu/g; and in the right kidney 1.00, 2.88 and 2.85 cfu/g. Respective median apoptosis of lymphocytes within the first 24 hours from induction of pancreatitis was 22.58, 23.45 and 24.19% (*P *= NS) whereas respective median apoptosis of monocytes was 41.02, 43.66 and 47.92% (*P *= NS) and of neutrophils was 76.84, 79.49 and 83.94% (*P *= 0.034).

## Conclusions

Survival in experimental necrotizing pancreatitis depends on the density of taurocholate. In spite of the existence of marginal differences in apoptosis of neutrophils occurring early during the course of the disease, it seems that apoptosis is not a major driver to death; instead, bacterial translocation seems to be the main route to death.

